# Thrombophilia‐Related Gene Polymorphisms and Recurrent Miscarriages in Lebanese Population: A Case‐Control Study

**DOI:** 10.1155/humu/5923585

**Published:** 2026-10-28

**Authors:** Ehssan Sharif-Askari, Khadija Atoui, Ola Srour, Linda Abou-Abbas, Mazen Fakih, Lamis Abou-Abbas, Zeinab Bazzi, Aline Milane, Antoine Barbari

**Affiliations:** ^1^ Biomedical Science Department School of Arts and Sciences Lebanese International University Tyre Campus Tyre Lebanon liu.edu.lb; ^2^ Nutrition and Food Technology Department School of Arts and Sciences Lebanese International University Nabatieh Campus Nabatieh Lebanon liu.edu.lb; ^3^ Faculty of Public and Health Sciences Islamic University of Lebanon Tyre Lebanon iul.edu.lb; ^4^ Medical Research Center Faculty of Medical Sciences Lebanese University Beirut Lebanon ul.edu.lb; ^5^ INSPECT-LB Institut National de Santé Publique Epidémiologie Clinique Et Toxicologie-Liban Beirut Lebanon; ^6^ Anesthesia and Reanimation Department Hospital Pierre Wertheimer Hospices Civils de Lyon Lyon France chu-lyon.fr; ^7^ Obstetrics and Gynecology Department Bahman Hospital Beirut Lebanon; ^8^ Department of Pharmaceutical Sciences School of Pharmacy Lebanese American University Beirut Lebanon lau.edu.lb; ^9^ Nephrology Department Rafik Hariri University Hospital Beirut Lebanon hcu.edu.lb

**Keywords:** cardiovascular disease, gene polymorphisms, inherited thrombophilia, recurrent pregnancy loss

## Abstract

**Background:**

Genes associated with cardiovascular diseases are strongly linked to idiopathic recurrent pregnancy loss. Because these genetic mutations often involve coagulation factors, anticoagulant therapy, particularly low‐molecular‐weight heparin, is increasingly encountered in obstetric practice in Lebanon for selected maternal and pregnancy‐related indications. However, data describing the prevalence and prescribing patterns of anticoagulant use among Lebanese obstetricians remain limited. In this multicenter retrospective case‐control study, 12 mutations within 10 CVD‐related genes in Lebanese women with a history of recurrent pregnancy loss were assessed to uncover further potential genetic links between these two conditions.

**Methods:**

Data were collected from a total of 117 women with recurrent pregnancy loss (≥ 2 pregnancy losses with the same partner before the 22nd week of gestation) and 38 women with a history of at least one successful pregnancy. Data were collected from two independent medical centers in Lebanon, Abou Jaoude Hospital and the Lebanese Diagnostic Medical Center, through medical‐record review and participant interviews. Twelve mutations were covered employing polymerase chain reaction and reverse‐hybridization techniques. Because of the variation in genotyping completeness across assays, the effective sample size for individual polymorphisms ranged from 27 to 38 controls and 78 to 117 cases.

**Results:**

Genetic analysis revealed a significant association between Factor V Leiden (OR = 0.31; *p* = 0.017), Factor XIII: V34L (OR = 0.3; *p* = 0.009), plasminogen activator inhibitor‐1: 4G/5G (OR = 0.3; *p* = 0.034), and lower odds of recurrent pregnancy loss. However, these associations did not remain significant after correction for multiple testing and should therefore be considered exploratory. No significant associations were observed for other evaluated polymorphisms, including methylenetetrahydrofolate reductase C677T and A1298C.

**Conclusion:**

Repeated pregnancy loss is multifaceted and not necessarily linked to coagulopathy‐related mutations; in the Lebanese population, certain polymorphisms showed nominal associations with lower odds of recurrent pregnancy loss that require confirmation in larger, independent studies, and no statistically significant association was observed between methylenetetrahydrofolate reductase mutations and recurrent pregnancy loss in the present study. These findings suggest that the routine presumption of a thrombophilic etiology in recurrent pregnancy loss may not be universally applicable. Future research should prioritize validating these genetic associations in larger, adequately powered cohorts and exploring the underlying biological mechanisms before any therapeutic implications are considered.

## 1. Introduction

Recurrent pregnancy loss (RPL) represents a distressing and emotionally challenging experience for the majority of affected women. Approximately 15%–25% of clinically recognized pregnancies end in miscarriage, and when unrecognized early losses are included, the miscarriage rate may be as high as 30%–60% of all pregnancies [1]. According to the American Society for Reproductive Medicine (ASRM), RPL is defined as the spontaneous loss of two or more pregnancies before 22 weeks of gestation, excluding confirmed molar or ectopic pregnancies [2], affecting 0.5%–3% of all fertile couples [3]. The ASRM explicitly notes that this 22‐week cutoff is consistent with the International Glossary of Infertility and Fertility Care and clarifies that pregnancy loss after 22 weeks is considered a stillbirth or fetal demise covered by dedicated ACOG/Society for Maternal–Fetal Medicine consensus guidelines [4]. Furthermore, the updated ASRM guidelines recognize very early pregnancy losses confirmed by blood or urine testing and clarify that these losses do not need to be consecutive to warrant evaluation [5]. Numerous instances of pregnancy loss arise from complications in the implantation process, which may not manifest clinically [6]. Considering these patients as a high‐risk obstetric population is essential, not only in the context of RPL management but also due to the specialized clinical attention required during subsequent pregnancies, since RPL mothers usually exhibit a predisposition towards preterm birth, an elevated incidence of C‐section, and an increased likelihood of postpartum hemorrhage [6].

The pathogenesis of RPL has been associated with many risk factors such as immune disorders, thrombophilia disorders, antiphospholipid syndrome, uterine anomalies, chromosomal abnormalities, coagulation factors, obesity, maternal age, infections, and polymorphisms in inflammatory mediators [7]. However, a substantial portion, up to 50%, of RPL cases remains idiopathic, posing a significant challenge to our current understanding of the underlying factors.

According to the World Health Organization (WHO), cardiovascular diseases (CVDs) are a group of disorders that affect the heart and blood vessels. In a meta‐analysis carried out by Oliver‐Williams et al. [8], they proved that patients with history of pregnancy loss were 45% more likely to develop coronary heart disease (CHD), a specific type of CVD, than healthy patients. Notably, individuals with a history of RPL exhibited nearly double the odds of developing CHD compared with those without RPL [8]. Although this long‐term epidemiological association does not necessarily imply shared genetic susceptibility, it suggests that common underlying vascular elements may exist between these two conditions. Specifically, both conditions may involve disruptions in the regulation of vascular processes crucial to both systemic endothelial health and early placentation [9].

Moreover, the association between mutations in CVD‐related genes and RPL has been extensively investigated. Although some studies have suggested a protective effect of certain variants [10, 11], others have pointed to their significant involvement in RPL pathogenesis [12, 13]. To provide a more balanced overview, recent systematic reviews and meta‐analyses have evaluated the role of thrombophilia‐related polymorphisms. For instance, a comprehensive 2023 systematic review and meta‐analysis by Wen et al. [14] evaluated the association of Factor V Leiden (FVL), prothrombin G20210A (Factor II [FII]), and MTHFR variants with RPL, highlighting the complex and sometimes conflicting nature of these genetic associations across different populations. Furthermore, the recent 2022 European Society of Human Reproduction and Embryology (ESHRE) guideline, which is based on extensive systematic reviews of the literature, concluded that the evidence linking FVL, FII, and MTHFR polymorphisms to early RPL remains inconsistent and highly population‐dependent [15].

Overall, studies have linked RPL with mutations in various coagulation and cardiovascular‐related factors, including FVL: *F5* G1691A [16], FII prothrombin: (*F2* G20210A) [14], angiotensin I converting enzyme (ACE) endothelial plasminogen activator inhibitor‐1 (PAI‐I) [17], *APO-E* [18, 19], *APO B* R3500Q [20, 21], methylenetetrahydrofolate reductase (*MTHFR* C677T) [22] and *MTHFR* A1298C [22], and platelet glycoprotein IIIa Pl A (1a/1b alleles) [23]. The rationale for investigating these specific cardiovascular‐related polymorphisms lies in their critical regulatory roles in endothelial function, the coagulation cascade, and fibrinolysis. Successful early pregnancy requires extensive trophoblast invasion and spiral artery remodeling, processes that depend on a tightly regulated, localized hemostatic and angiogenic balance at the maternal–fetal interface [6, 9]. Genetic variants that predispose individuals to cardiovascular endothelial dysfunction or altered thrombotic and fibrinolytic states, such as those affecting Factor V (FV), FII, Factor XIII (FXIII), PAI‐1, and MTHFR, were hypothesized to similarly disrupt this delicate placental vascular remodeling [11, 14, 24]. Therefore, these specific CVD‐related variants were selected to evaluate their potential biological impact on early placentation and RPL.

To date, research in Lebanon exploring the link between mutations in CVD‐related genes and RPL remains limited. However, study performed by El Achi et al. [25] shows a “strong relationship” between *MTHFR* (C677T homozygosity, A1298C homozygosity, and compound heterozygosity for C677T and A1298C) and FII (heterozygosity for G20210A) mutations and RPL. Yet, the controls used in this study to demonstrate their findings included biological males in the samples [26–31]. Despite the lack of robust local genetic evidence, empirical anticoagulant therapy, particularly low‐molecular‐weight heparin, is increasingly encountered in Lebanese obstetric practice for women with RPL. However, data on the actual prevalence of CVD‐related mutations and anticoagulant prescribing patterns among Lebanese obstetricians remain limited [32, 33]. Against this background, the present study provides an updated, multicenter, female‐focused, and broader genetic assessment, generating population‐specific exploratory evidence on thrombophilia‐related polymorphisms and RPL among Lebanese women.

In the current study, we investigated the associations between 12 mutations across 10 CVD‐related genes and RPL among Lebanese women.

## 2. Materials and Methods

### 2.1. Patients and Data Collection

The current study is a retrospective, multicenter case‐control analysis with data collected from two independent medical centers in Lebanon: The Obstetrics and Gynecology Department of Abou Jaoude Hospital and the Lebanese Diagnostic Medical Center (LDC) Laboratory. The study obtained ethical approval from LIU Institution Review Board under the number of LIUIRB‐230226‐ESA‐241 and adhered to the principles of the Declaration of Helsinki. Researchers and fieldworkers conducted the study with a strong emphasis on privacy and confidentiality, ensuring that all patient data used in the research were anonymized and deidentified.

A total of 117 women with RPL, defined by a history of at least two pregnancy losses with the same partner and referred to medical laboratories were selected as the case group. Exclusion criteria included previous thromboembolic events, known fetal demise, planned pregnancy termination, maternal antiphospholipid, lupus anticoagulant or anticardiolipin antibodies, or any anatomical abnormalities likely to contribute to pregnancy loss. A total of 38 women with a history of at least one successful pregnancy, devoid of any pregnancy failures, and without personal or familial occurrences of inherited thrombophilia were used as a control group.

Data were collected similarly for both case and control groups between February 27th, 2023, and April 22nd, 2024, by a team that did not include the researchers and secured verbal consent to participate in the study. This consent was documented and signed off by a witness. Each phone interview with eligible participants lasted approximately 10 min. Participation in this study was absolutely voluntary, and individuals had the complete right to stop the interview once they needed. If an individual decided not to answer further questions, the interview was terminated and gathered data were discarded.

During the first part, the investigators obtained baseline characteristics such as date of birth, self‐reported weight, and height. Additionally, respondents provided details on their obstetric history, including the number of gravida (confirmed pregnancies), partum (number of births after 20 weeks of gestation), abortions (termination of pregnancy), and consecutive pregnancy losses since their first marriage. The second part involved extracting data from medical records, focusing on the age of pregnant women during their initial clinic or laboratory visit. In addition, laboratory results from genetic CVD panel of testing were also recorded.

### 2.2. Genetic Analysis

Genetic testing was performed at two independent diagnostic centers using distinct analytical platforms. Genomic DNA was isolated from whole blood samples collected in EDTA tubes using standard laboratory procedures.

At Abou Jaoude Hospital, genetic analysis for the full panel of 12 CVD‐related mutations employed a combined approach. First, real‐time PCR was performed for the three most prevalent mutations—FVL (G1691A), MTHFR (C677T), and MTHFR (A1298C)—using specific LightMix Kits (TIB Molbiol, Berlin, Germany) on the Roche LightCycler 96 System (Roche Diagnostics, Basel, Switzerland). Genotypes were determined based on fluorescence signals and threshold cycle (CT) values. Second, a reverse‐hybridization strip assay was used for the remaining nine mutations (prothrombin G20210A, FV R2 H1299R, FXIII V34L, PAI‐1 4G/5G, *β*‐fibrinogen −455G>A, GPIIIa HPA 1a/b, ACE I/D, ApoE, and ApoB R3500Q) using the CVD StripAssay (ViennaLab, Vienna, Austria). This assay involves PCR amplification followed by reverse‐hybridization of biotinylated DNA to allele‐specific oligonucleotide probes on test strips. Interpretation relied on the presence or absence of specific visual bands. For ApoE, this process identified six possible isoforms (E2/2, E3/3, E4/4, E2/3, E2/4, and E3/4).

At the Lebanon Medical Diagnostic Center, targeted quantitative real‐time PCR (qRT‐PCR) was performed for four specific mutations: FII (prothrombin G20210A), FVL (G1691A), MTHFR C677T, and MTHFR A1298C. This assay utilized multiplex PCR reagents from Anatolia Geneworks (Turkey) based on the 5 ^′^ exonuclease activity of Taq Polymerase with dual‐labeled fluorescent hydrolysis probes. Genotypes (wild‐type, heterozygous, or homozygous mutant) were automatically interpreted by the real‐time PCR instrument software based on CT values. Although data for four genetic mutations FVL: G1691A, prothrombin (FII): G20210A, (*MTHFR*): C677T, and (*MTHFR*): A1298C were available for all patients, information for the remaining eight mutations was confined to results obtained from Abou Jaoude Hospital.

### 2.3. Statistical Analysis

Data analyses were conducted using the statistical software SPSS Version 23.0. Descriptive statistics, comprising means with standard deviation for normally distributed continuous variables and medians with ranges for skewed continuous variables, were reported. Categorical variables underwent analysis using frequencies and percentages. Baseline and clinical characteristics differences between cases and controls were evaluated through the chi‐square test or Fisher′s exact test for categorical variables, as appropriate, and the independent samples *t*‐test or Mann–Whitney test for continuous variables depending on data distribution.

Binary logistic regression models were used to evaluate the association between each of the 12 gene polymorphisms and RPL, with case/control status as the dependent variable and genotype as the independent variable (wild‐type genotype as the reference category). Models were fit separately for each locus (univariate). Odds ratios (ORs) and corresponding 95% confidence intervals (CIs) were calculated for each genetic model (codominant, dominant, and recessive) to examine the strength and direction of these associations.

Hardy–Weinberg equilibrium was assessed for each polymorphism among controls using an exact test [34], given the small sample size and presence of sparse genotype categories for several loci; the standard asymptotic chi‐square test was also computed for comparison. A *p* value < 0.05 under the exact test was considered indicative of deviation from Hardy–Weinberg expectations.

To address the issue of multiple comparisons resulting from the evaluation of 12 polymorphisms, Benjamini–Hochberg false discovery rate (BH‐FDR) correction was additionally applied. Adjusted *p* values were reported alongside nominal *p* values.

Post hoc power analyses were performed for polymorphisms showing statistically significant or suggestive associations, using the observed genotype distributions, the sample size available for each polymorphism (27–38 controls and 78–117 cases, reflecting variation in genotyping completeness across assays), and a two‐sided significance level of 0.05.

A significance level of *p* < 0.05 was considered for statistical significance.

## 3. Results

Baseline characteristics of both cases and controls are shown in Table 1. A total of 155 patients were enrolled in the study, consisting of 117 cases with unexplained pregnancy loss and 38 healthy women with uncomplicated pregnancies. The mean age of the participants was 34.0 years, with a standard deviation of 6.3 years. Notably, there were no statistically significant differences in age between the cases and controls; mean ages were 34.4 and 32.6 for cases and controls, respectively (*p* value = 0.129). Similarly, there were no significant differences observed between cases and controls concerning BMI, indicating a well‐matched study cohort in terms of age and BMI.

**Table 1:** Baseline characteristics of the study participants.

Variables	Control (*n* = 38)	Cases (*n* = 117)	*p*
Age (mean ± SD)	32.6 ± 6.5	34.4 ± 6.2	0.129
BMI (mean ± SD)	24.7 ± 5.3	25.5 ± 5.2	0.696

*Note:* Statistical analysis conducted using Student *t*‐test, *p* value < 0.05 is considered significant.

Abbreviations: BMI, body mass index; *n*, frequency; SD, standard deviation.

### 3.1. Obstetric History of Cases and Controls

Table 2 depicts the obstetric history of cases and controls. Compared with controls, women with a history of unexplained RPL were significantly more likely to be of higher gravidity, pregnancy loss, and lower parity.

**Table 2:** Obstetric history of cases and control.

Variables	Control median (range)	Cases median (range)
Number of gravida	1 (1–4)	4 (2–13)
Number of partum	1 (1–4)	1 (0–6)
Number of pregnancy loss	0 (0)	2 (2–12)

*Note:* Number of gravida: total number of times a woman has been pregnant, regardless of the outcome. Number of partum: number of times a woman has given birth to a viable offspring. Number of pregnancy loss: number of pregnancies that did not result in a live birth.

### 3.2. Genotyping Results

Table 3 displays the genotyping results showing differences between the cases and controls. The results for the FVL indicate that homozygous variant genotypes were present in 23.7% of controls and 8.5% of cases (OR = 0.31; 95% CI between 0.113 and 0.817; *p* value = 0.020). In the dominant model (combining GA and AA genotypes), no statistical significance was found (OR = 0.476; 95% CI between 0.202 and 1.120; *p* value = 0.089). In the recessive model (considering only the AA genotype), a significant association with lower odds of RPL was found (OR = 0.301; 95% CI between 0.112 and 0.810; *p* value = 0.018).

**Table 3:** Genotype distributions of the Factor V, prothrombin, and methylenetetrahydrofolate reductase.

Genetic mutations	Control *n*(%)	Cases *n*(%)	OR 95% (CI)	*p*∗
Factor V (Leiden): G1691A				
Wild type GG	27 (71.7)	98 (83.8)	1.00	
Heterozygous (carrier) GA	2 (5.3)	9 (7.7)	1.24 (0.253–6.081)	0.791
Homozygous (mutant) AA	9 (23.7)	10 (8.5)	0.31 (0.113–0.817)	0.020
FVL dominant				
Wild type	27 (71.7)	98 (83.8)	1.00	
Heterozygous (carrier) and homozygous (mutant)	11 (28.9)	19 (16.2)	0.476 (0.202–1.120)	0.089
FVL recessive				
Wild type and heterozygous (carrier)	29 (76.3)	107 (91.5)	1.00	
Homozygous (mutant)	9 (23.7)	10 (8.5)	0.301 (0.112–0.810)	0.017
Prothrombin (FII): G20210A				
Wild type GG	34 (89.5)	98 (83.8)	1.00	
Heterozygous (carrier) GA	4 (10.5)	13 (11.1)	1.128 (0.344–3.694)	0.843
Homozygous (mutant) AA	0 (0)	6 (5.8)	—	0.999
Prothrombin (FII) dominant				
Wild type	34 (89.5)	98 (83.8)	1.00	
Heterozygous (carrier) and homozygous (mutant)	4 (10.5)	19 (16.9)	1.648 (0.524–5.187)	0.393
Prothrombin (FII) recessive				
Wild type and heterozygous (carrier)	38 (100)	111 (94.2)	1.00	
Homozygous (mutant)	0 (0)	6 (5.8)	—	0.999
Methylenetetrahydrofolate reductase (MTHFR): C677T				
Wild type CC	18 (47.3)	55 (47.0)	1.00	
Heterozygous (carrier) CT	12 (31.6)	21 (18.0)	0.573 (0.236–1.390)	0.218
Homozygous (mutant) TT	8 (21.1)	41 (35.0)	1.677 (0.665–4.233)	0.274
MTHFR C677 dominant				
Wild type	18 (47.3)	55 (47.0)	1.00	
Heterozygous (carrier) and homozygous (mutant)	20 (52.7)	62 (52.9)	1.015 (0.487–2.112)	0.969
MTHFR C677 recessive				
Wild type and heterozygous (carrier)	30 (78.9)	76 (65.0)	1.00	
Homozygous	8 (21.1)	41 (35.0)	2.023 (0.850–4.816)	0.111
MTHFR: A1298 C				
Wild type AA	16 (43.2)	41 (35.7)	1.00	
Heterozygous (carrier) AC	15 (40.5)	37 (32.2)	1.020 (0.419–2.214)	0.929
Homozygous (mutant) CC	6 (16.2)	32 (32.2)	2.407 (0.852–6.796)	0.097
MTHFR: A1298 C dominant				
Wild type	16 (43.2)	41 (35.7)	1.00	
Heterozygous (carrier) and homozygous (mutant)	21 (54.5)	69 (62.4)	1.375 (0.647–2.293)	0.408
MTHFR: A1298 C recessive				
Wild type and heterozygous (carrier)	31 (81.8)	78 (68.3)	1.00	
Homozygous (mutant)	6 (18.2)	32 (31.7)	2.451 (0.941–6.386)	0.067

*Note:* Genotype distributions of the Factor V Leiden (FVL): G1691A, prothrombin (FII): G20210A, methylenetetrahydrofolate reductase (MTHFR): C677T, and MTHFR: A1298C. OR and 95% CI derived from univariate binary logistic regression.

Abbreviations: %, percentage; CI, confidence interval; *n*, frequency; OR, odds ratio.

∗*p* value ˂ 0.05 is considered significant.

For the prothrombin gene mutation (G20210A), no significant differences in genotype distributions were found between the control group (10.5% heterozygous and 0% homozygous) and cases (11.1% heterozygous and 5.8% homozygous) with a nonstatistically significant difference. In addition, no statistically significant associations with RPL in either the dominant or recessive models were found (*p* value > 0.05). For the *MTHFR* C677T and *MTHFR* A1298C polymorphisms, the results suggest no significant association with RPL in either the dominant or the recessive models.

Furthermore, results in Table 4 indicate that heterozygous FXIII: V34L genotype was present in 29.1% of cases and 59.3% of controls (OR = 0.288; 95% CI between 0.116 and 0.733; *p* value = 0.007). In the dominant model (combining GA and AA genotypes), OR was 0.3 with a 95% CI of 0.121 and 0.742, *p* value = 0.009 suggesting an association between the FXIII V34L variant and lower odds of RPL. Nevertheless, no significant association was found in the recessive model with RPL. For plasminogen activator inhibitor (PAI‐1): The homozygous 4G/4G genotype was observed in 12 individuals (44.4%) in the control group and 18 individuals (22.7%) in the case group (*p* value = 0.046). When considering the dominant genotype (heterozygous carriers and homozygous mutants), 25 individuals (92.6%) in the control group and 63 individuals (79.7%) in the case group exhibited this combined genotype, but no significant difference was found (*p* value = 0.142). For the recessive genotype (homozygous mutants), 12 individuals (44.4%) in the control group and 18 individuals (22.7%) in the case group were identified (*p* value = 0.034), indicating an association between the recessive PAI‐1 4G/4G genotype model and lower odds of RPL. For the remaining CVD genes FV (R2) H1299R, *β*‐fibrinogen −455G>A, platelet glycoprotein IIIa (GPIIIA) HPA 1: a/b, ACE: insertion/deletion, and apolipoproteins (Apo E), no statistically significant associations were observed.

**Table 4:** Genotype distributions of other specific CVD‐genes.

Genetic mutations	Control *n*(%)	Cases *n*(%)	OR 95% (CI)	*p*∗
Factor V (R2): H1299R				
Wild type	21 (77.8)	64 (82.1)	1.00	
Heterozygous (carrier)	6 (22.2)	14 (17.9)	0.766 (0.261–2.245)	0.627
Homozygous (mutant)	0 (0)	0 (0)		
Factor V (R2): H1299R dominant				
Wild type	21 (77.8)	64 (82.1)	1.00	
Heterozygous (carrier) and homozygous (mutant)	6 (22.2)	14 (17.9)	0.82 (0.282–2.385)	0.716
Factor V (R2): H1299R recessive				
Wild type and heterozygous (carrier)	27 (100)	78 (100)	—	
Homozygous (mutant)	0 (0)	0 (0)	—	
Factor XIII: V34L				
Wild type	11 (40.7)	55 (69.6)	1.00	
Heterozygous (carrier)	16 (59.3)	23 (29.1)	0.288 (0.116–0.733)	0.007
Homozygous (mutant)	0 (0)	1 (1.3)	—	
Factor XIII: V34L dominant				
Wild type	11 (40.7)	55 (69.6)	1.00	
Heterozygous (carrier) and homozygous (mutant)	16 (59.3)	24 (30.4)	0.3 (0.121–0.742)	0.009
Factor XIII: V34L recessive				
Wild type and heterozygous (carrier)	27 (100)	78 (98.7)	—	
Homozygous (mutant)	0 (0)	1 (1.3)	—	
*β*‐Fibrinogen: −455G>A				
Wild type	14 (51.9)	47 (59.5)	1.00	
Heterozygous (carrier)	13 (48.1)	28 (35.4)	0.642 (0.264–1.559)	0.327
Homozygous (mutant)	0 (0)	4 (5.1)	—	
*β*‐Fibrinogen: −455G>A dominant				
Wild type	14 (51.9)	47 (59.5)	1.00	
Heterozygous (carrier) and homozygous (mutant)	13 (48.1)	32 (39.1)	0.733 (0.305–1.765)	0.489
*β*‐Fibrinogen: −455G>A recessive				
Wild type and heterozygous (carrier)	27 (100)	75 (94.9)		
Homozygous (mutant)	0 (0)	4 (5.1)	—	
Plasminogen activator inhibitor (PAI‐1): 4G/5G				
Wild type	2 (7.4)	16 (20.3)	1.00	
Heterozygous (carrier)	13 (48.1)	45 (57.0)	0.433 (0.088–2.131)	0.303
Homozygous (mutant)	12 (44.4)	18 (22.7)	0.188 (0.036–0.968)	0.046
Plasminogen activator inhibitor (PAI‐1): 4G/5G dominant				
Wild type	2 (7.4)	16 (20.3)	1.00	
Heterozygous (carrier) and homozygous (mutant)	25 (92.6)	63 (79.7)	0.315 (0.067–1.471)	0.142
Plasminogen activator inhibitor (PAI‐1): 4G/5G recessive				
Wild type and heterozygous (carrier)	15 (55.6)	61 (77.3)	1.00	
Homozygous (mutant)	12 (44.4)	18 (22.7)	0.369 (0.147–0.929)	0.034
Platelet glycoprotein IIIa (GPIIIA) HPA 1: a/b				
Wild type	16 (59.3)	52 (65.8)	1.00	
Heterozygous (carrier)	11 (40.7)	24 (30.4)	0.671 (0.271–1.663)	0.389
Homozygous (mutant)	0 (0)	3 (3.8)	—	
Platelet glycoprotein IIIa (GPIIIA) HPA 1: a/b dominant				
Wild type	16 (59.3)	52 (65.8)	1.00	
Heterozygous (carrier) and homozygous (mutant)	11 (40.7)	27 (34.2)	0.755 (0.308–1.853)	0.540
Platelet glycoprotein IIIa (GPIIIA) HPA 1: a/b recessive				
Wild type and heterozygous (carrier)	27 (100)	76 (96.2)		
Homozygous (mutant)	0 (0)	3 (3.8)	—	
Angiotensin I‐converting enzyme: insertion/deletion				
Wild type	15 (55.6)	29 (37.2)	1.00	
Heterozygous (carrier)	9 (33.3)	37 (47.4)	0.483 (0.118–1.98)	0.312
Homozygous (mutant)	3 (11.1)	12 (15.4)	1.028 (0.239–4.425)	0.971
Angiotensin I‐converting enzyme: insertion/deletion dominant				
Wild type	15 (55.6)	29 (37.2)	1.00	
Heterozygous (carrier) and homozygous (mutant)	12 (44.4)	49 (62.8)	2.155 (0.888–5.229)	0.089
Angiotensin I‐converting enzyme: insertion/deletion recessive				
Wild type and heterozygous (carrier)	24 (88.9)	66 (84.6)	1.00	
Homozygous (mutant)	3 (11.1)	12 (15.4)	1.455 (0.378–5.603)	0.586
Apolipoproteins (Apo B): R3500Q				
Wild type	27 (100)	79 (100)		
Heterozygous (carrier)	0 (0)	0 (0)	NA	
Homozygous (mutant)	0 (0)	0 (0)	NA	
Apolipoproteins (Apo E)				
Wild type	23 (85.2)	66 (83.5)	1.00	
Heterozygous (carrier)	4 (14.8)	13 (16.5)	1.133 (0.335–3.825)	0.841
Homozygous (mutant)	0 (0)	0 (0)	—	
Apolipoproteins (Apo E) dominant				
Wild type	23 (85.2)	66 (83.5)	1.00	
Heterozygous (carrier) and homozygous (mutant)	4 (14.8)	13 (16.5)	1.133 (0.335–3.825)	0.841
Apolipoproteins (Apo E) recessive				
Wild type and heterozygous (carrier)	27 (100)	79 (100)		
Homozygous (mutant)	0 (0)	0 (0)		

*Note:* Genotype distributions of the Factor V (R2): H1299R, Factor XIII: V34L, *β*‐fibrinogen: −455G>A, plasminogen activator inhibitor (PAI‐1): 4G/5G, platelet glycoprotein IIIa (GPIIIA) HPA 1: a/b, angiotensin I‐converting enzyme: insertion/deletion, apolipoproteins (Apo E). OR and 95% CI derived from univariate binary logistic regression.

Abbreviations: %, percentage; CI, confidence interval; *n*, frequency; OR, odds ratio.

∗*p* value ˂ 0.05 is considered significant.

### 3.3. Statistical Power

Post hoc power analysis was performed for the three polymorphisms reaching nominal significance. The study had 62.0% power to detect the observed FVL recessive association (38 controls and 117 cases), 75.3% power for the FXIII V34L association (27 controls and 79 cases), and 54.9% power for the PAI‐1 4G/5G recessive association (27 controls and 79 cases). A control group of approximately 49–58 participants would have been required to achieve 80% power for these effect sizes at the observed case: control ratio.

### 3.4. Hardy–Weinberg Equilibrium and Correction for Multiple Comparisons

Genetic analysis for HWE showed that all polymorphisms were consistent with HWE expectations except FVL G1691A (exact *p* < 0.0001) (Table S1). To account for the twelve polymorphisms evaluated, Bonferroni and BH‐FDR corrections were applied across all 32 statistically valid comparisons; none of the six nominally significant associations remained significant after correction (Bonferroni *p* = 0.224–1.000; FDR *q* = 0.144–0.245) (Table S2).

## 4. Discussion

In elucidating the connection between CVD‐related genetic polymorphisms and RPL, our study provides additional evidence regarding the complex and population‐specific nature of these associations. Contrary to previous assumptions reported in several studies [12, 13, 35], our findings unveil associations between selected polymorphisms and lower odds of RPL. These findings should be interpreted cautiously, as they may reflect population‐specific genetic characteristics rather than direct protective biological effects. An important strength of our study is the selection of women as controls, allowing comparison within the same biological context. However, the relatively limited sample size and the imbalance between cases and controls should also be considered when interpreting the findings.

Numerous studies have explored the link between mutations in coagulation factors and RPL. The majority of these studies focus specifically on coagulation factors such as FV, FII, FXIII, and PAI‐1. Despite these investigations, conflicting findings persist across different ethnic groups. Further exploration of their roles remains essential for a comprehensive understanding of the genetic factors contributing to RPL.

Coagulation (FV) is pivotal in orchestrating blood coagulation, proving a dual role by performing both procoagulant and anticoagulant functions [36]. FVL is a genetic disorder characterized by impaired anticoagulant response to activated protein C (APC) [37]. The relation between FVL mutation and RPL has been an open debate for many years, with studies reporting conflicting findings [35, 38, 39]. However, in our cohort, the homozygous FVL genotype was observed more frequently among controls than cases, resulting in an association with lower odds of RPL. FVL clinical impact on early pregnancy outcomes remains highly heterogeneous across different populations. In line with our findings, several large cohort studies and meta‐analyses have reported no significant association between FVL and RPL, suggesting that its role in early gestational failure is not consistently harmful [40–42]. Similarly, prospective cohort data have demonstrated that maternal heterozygous FVL carriage is not associated with an increased risk of pregnancy loss or other adverse obstetric outcomes, with comparable rates observed between carriers and noncarriers [43]. From a mechanistic perspective, although early implantation and placentation require a tightly regulated hemostatic balance, there is no established experimental or clinical evidence to suggest that the FVL mutation confers a biological advantage or “protective effect” during early gestation. The biological mechanisms underlying the heterogeneous associations between FVL and RPL remain incompletely understood. Differences in genetic background, allele distribution, and population‐specific coagulation profiles may contribute to the variability observed across populations. This concept is indirectly supported by observations that FVL is more consistently associated with complications arising later in pregnancy, such as placental insufficiency and preeclampsia, rather than early miscarriage. Therefore, the association observed in our Lebanese cohort likely reflects a context‐dependent genetic–physiological interaction rather than a universal biological mechanism, warranting validation in larger, multiethnic studies with longitudinal coagulation profiling.

Some papers reported the impact of coagulation FII also known as prothrombin in RPL [12, 13, 44]. Thrombin converts fibrinogen into fibrin, activates platelets and increases endothelial permeability thereby stopping blood loss at the site of injury [45]. Often, patients experience specific variations in their genetic code known as single‐nucleotide polymorphisms (SNPs), which are frequently associated with various degrees of bleeding tendencies, and the G20210A mutation found in the 3 ^′^ untranslated region of the *F2* gene is an important risk factor for thrombophilia in these cases [46]. In our study, FII polymorphism was not significantly associated with RPL, similar to previous study′s results [12, 13].

MTHFR mutations, represented by the common variants 677C>T and 1298A>C, are widespread globally, where 60%–70% of people have at least one of these genetic variations with a total of 10% having homozygous or compound heterozygous combinations [47]. Both mutations are shown to alter the enzymatic activity and homocysteine concentrations [48], an emerging CVD risk factor [49], since homocysteine may cause irritation in blood vessels and is considered a risk factor for blood clots. Our results showed no association between *MTHFR* mutations and RPL. The associative role of *MTHFR* mutations in RPL is a subject of ongoing debate. The reason why many studies supported the association between *MTHFR* mutation and RPL is its role in folate metabolism and the importance of folate during pregnancy [50]. During pregnancy, folate plays a crucial role in the growth and development of the fetus, and its deficiency has been linked to abnormalities in both mothers, leading to conditions like anemia and peripheral neuropathy, as well as in fetuses, contributing to the occurrence of congenital abnormalities [50]. Although numerous studies suggest a correlation, a substantial body of research, including our current article, contradicts the correlation, finding no significant association between *MTHFR* mutations and RPL [51–53]. There is neither clear pathogenesis nor pathophysiology as to why *MTFHR* mutations may cause RPL. Therefore, current evidence does not support the use of anticoagulant therapy solely based on the presence of MTHFR polymorphisms in women with RPL. Management decisions should consider established clinical indications rather than genetic status alone. Given the lack of significant association between MTHFR polymorphisms and RPL in our cohort, our findings do not support the routine use of anticoagulant therapy solely based on the presence of MTHFR variants, especially since certain oral anticoagulants cross the placenta and have been associated with central nervous system anomalies, fetal hemorrhage, facial abnormalities, and potential mental challenges [54]. Moreover, we highly recommend that pregnant women with MTHFR mutations continue taking folic acid, as it has demonstrated positive results in improving coagulation status by preventing blood clot formation and abnormal coagulation [55, 56]. However, definitive clinical recommendations regarding folic acid supplementation versus anticoagulation require evidence from randomized controlled trials, and management decisions should be guided by established clinical risk factors rather than genetic polymorphism status alone.

Another important factor is FXIII. It is the final factor in the coagulation cascade, consisting of subunits FXIIIA and FXIIIB, uniquely stabilizes blood clots by cross‐linking fibrin, enhancing clot density, stiffness, and contributing to platelet‐dependent clot retraction, wound healing, and tissue repair [57]. In the context of the FXIII V34L mutation and its relation to RPL, other studies have corroborated our current findings, as they showed a higher frequency of the mutated allele in the control group compared with the study group [12, 13]. Nevertheless, the biological mechanism underlying this association remains unclear, and the observed relationship should be interpreted cautiously.

Most researches have been linking PAI‐1 polymorphisms to PRL [24, 58]. Interestingly, our findings differed from some previous reports, as the PAI‐1 4G/5G polymorphism was observed more frequently among controls than cases. PAI‐1 4G/5G polymorphism was associated with lower odds of RPL, where it was present in 44.4% of control group against 22.7% of studied group. PAI‐1 is a member of the serine protease inhibitors (serpins) family that plays a significant role in regulating the plasminogen/plasmin system [59] and its polymorphism significantly triggers fibrin deposit, leading to thrombosis. The prevalent polymorphism in the *PAI-1* gene involves an insertion‐deletion mutation at Position −675, often denoted as 4G/5G [60]. Assuming that having multiple thrombophilic mutations in a single individual could cumulatively increase the risk of miscarriage, certain publications have examined specific combinations of alterations [61, 62]. In previous studies, researchers have not found any specific combination of alterations that could offer protection. Given the limited sample size and the loss of statistical significance after multiple‐testing correction, these findings should not be interpreted as evidence of a protective genetic combination. Rather, they highlight the need for adequately powered studies to determine whether population‐specific patterns of thrombophilia‐related variants exist among Lebanese women with RPL.

Similarly, regarding FXIII V34L and PAI‐1 4G/5G polymorphisms, no clear biological mechanism or pathophysiological basis exists to explain a protective effect against RPL. In fact, these variants are generally considered to alter fibrin clot structure or fibrinolytic activity in ways that would theoretically increase, rather than decrease, thrombotic risk. Therefore, the unexpected “protective” associations observed for these polymorphisms in our study are highly likely to be spurious findings driven by the limited sample size, rather than genuine biological effects.

Several alternative explanations must be considered for these unexpected findings. First, the limited sample size, particularly the small number of controls, reduces statistical power and increases the likelihood of Type I errors (false positives). Second, technical issues or batch effects related to genotyping across different sites cannot be entirely ruled out, although sensitivity analyses were performed. Third, the case group is highly heterogeneous; RPL is a multifactorial condition, and our cohort included women with varying numbers of prior losses, different gestational ages at the time of loss, and both primary and secondary RPL. This heterogeneity may dilute true genetic effects or introduce confounding.

Several limitations should be considered when interpreting our findings. First, the relatively small sample size, particularly the limited number of control participants, and the resulting imbalance between cases and controls, may have reduced statistical power to detect modest genetic effects and increased the risk of chance findings. Although post hoc power analyses were performed for the observed associations, the study may remain underpowered for smaller effect sizes, and validation in larger independent cohorts is required. Second, multiple genetic polymorphisms were evaluated, increasing the possibility of false‐positive findings due to multiple testing. Although we applied a BH‐FDR correction, none of the nominally significant associations remained significant after adjustment, indicating that the observed effects are likely due to chance. Therefore, the reported associations should be considered exploratory and interpreted cautiously until confirmed in larger populations. Third, the case group included both primary and secondary RPL in the same analysis, which may introduce heterogeneity, as the etiologies for these two categories can differ. Fourth, there was incomplete genetic data for several polymorphisms, as data for 8 of the 12 mutations were only available from a single recruitment site, reducing the sample size for those specific analyses. In addition, deviations from Hardy–Weinberg equilibrium observed for some variants may reflect sampling variability, population characteristics, or other methodological factors and should be considered when interpreting these genetic associations. Fifth, the study population consisted exclusively of Lebanese women, which may limit the generalizability of the findings to other ethnic groups or populations with different genetic backgrounds. Finally, genotyping was performed using two laboratory methods at two recruitment sites (Abou Jaoude Hospital and LDC Lab), raising the possibility of site‐related methodological bias. Although genotype distributions differed between sites, sensitivity analyses were performed to evaluate this potential source of bias. Genotype‐by‐site interaction analyses showed no statistically significant interactions for the evaluated polymorphisms, and adjustment for recruitment site resulted in minimal changes in the estimated genotype associations. Nevertheless, because the study was not originally designed to evaluate interlaboratory variability and the sample size limits the ability to detect small interaction effects, replication using a single genotyping platform and standardized methodology in larger cohorts is recommended.

## 5. Conclusion

RPL is multifaceted and not necessarily associated with mutations that result in coagulopathies. In this case‐control study, we assessed the potential genetic links between 10 CVD‐related genes and RPL in Lebanese women. Although in previous studies, FVL, FXIII: V34L, and PAI‐1: 4G/5G mutations have been shown to be strongly correlated with RPL, our findings did not demonstrate an increased susceptibility to RPL associated with these variants. Instead, some polymorphisms were observed more frequently among controls, resulting in associations with lower odds of RPL in the studied Lebanese population. However, reflecting the observational nature of this study and its limitations, including a relatively small sample size and the loss of statistical significance after correction for multiple comparisons, these protective associations must be interpreted as preliminary findings. They require confirmation in larger, independent, and adequately powered studies rather than being viewed as established biological effects. Moreover, no statistically significant association was observed between *MTHFR* polymorphisms and RPL in the present study. These findings are consistent with growing evidence questioning the clinical relevance of MTHFR variants as independent determinants of pregnancy loss. Therefore, management decisions for women with RPL should be guided by established clinical risk factors rather than genetic polymorphism status alone. Future research should prioritize validating the observed genetic associations in larger, adequately powered cohorts and exploring the underlying biological mechanisms before any therapeutic implications are considered. A deeper understanding of the complex genetic, environmental, and lifestyle factors contributing to RPL will ultimately help guide evidence‐based clinical practices and improve outcomes for affected women.

## Author Contributions

Antoine Barbari and Ehssan Sharif‐Askari conceptualized the project idea. Ehssan Sharif‐Askari and Khadija Atoui performed the literature review. Ola Srour, Mazen Fakih, Lamis Abou‐Abbas, Aline Milane, and Zeinab Bazzi formulated the questionnaire and collected the data. Linda Abou‐Abbas analyzed the data and contributed to the writing and interpretation of the findings. The first draft of the manuscript was written by Khadija Atoui. The drafted article was critically revised by Ola Srour, Mazen Fakih, Linda Abou‐Abbas and Ehssan Sharif‐Askari. Antoine Barbari, and Ehssan Sharif‐Askari supervised the project and critically reviewed the paper for important intellectual content. The final version was edited by Ehssan Sharif‐Askari.

## Funding

No funding was received for this manuscript.

## Disclosure

All authors read and approved the final manuscript.

## Conflicts of Interest

The authors declare no conflicts of interest.

## Supporting information


**Supporting Information** Additional supporting information can be found online in the Supporting Information section. Table S1 details the Hardy–Weinberg equilibrium (HWE) assessment for the control group, reporting the number of successfully genotyped individuals, genotype counts, minor allele frequencies, and both chi‐square and exact *p* values to confirm HWE consistency across all evaluated loci. Table S2 presents the genetic association results evaluated under multiple inheritance models (codominant, dominant, and recessive). For each polymorphism, it lists the unadjusted raw *p* values alongside multiple‐testing corrected values, specifically the Bonferroni‐adjusted *p* values and BH‐FDR *q* values, to clearly identify nominally significant associations.

## Data Availability

The data that support the findings of this study are available on request from the corresponding author. The data are not publicly available due to privacy or ethical restrictions.
