# Supplementary material for: Thrombophilia‐Related Gene Polymorphisms and Recurrent Miscarriages in Lebanese Population: A Case‐Control Study
Source: Hum Mutat. 2026 Oct 28;2026:5923585. doi: 10.1155/humu/5923585 (PMC13717292; doi:10.1155/humu/5923585)
Supplement: Supplementary file 1 [file HUMU-2026-5923585-s001.docx]

## **Table S1**

| **Locus** | **n (control)** | **Genotype counts (AA / Aa / aa)** | **Minor allele frequency** | **χ² p-value** | **Exact p-value** | **HWE conclusion** |
| --- | --- | --- | --- | --- | --- | --- |
| Factor V Leiden G1691A | 38 | 27 / 2 / 9 | 0.263 | <0.0001 | <0.0001 | Deviates |
| Prothrombin G20210A | 38 | 34 / 4 / 0 | 0.053 | 0.732 | 1.000 | Consistent |
| MTHFR C677T | 38 | 18 / 12 / 8 | 0.368 | 0.048 | 0.077 | Consistent |
| MTHFR A1298C | 37 | 16 / 15 / 6 | 0.365 | 0.446 | 0.484 | Consistent |
| Factor V R2 (H1299R) | 27 | 21 / 6 / 0 | 0.111 | 0.516 | 1.000 | Consistent |
| Factor XIII V34L | 27 | 11 / 16 / 0 | 0.296 | 0.029 | 0.060 | Consistent |
| β-Fibrinogen −455G>A | 27 | 14 / 13 / 0 | 0.241 | 0.099 | 0.283 | Consistent |
| PAI-1 4G/5G | 27 | 2 / 13 / 12 | 0.685 | 0.547 | 1.000 | Consistent |
| GPIIIA HPA1 a/b | 27 | 16 / 11 / 0 | 0.204 | 0.184 | 0.549 | Consistent |
| ACE Insertion/Deletion | 27 | 15 / 9 / 3 | 0.278 | 0.379 | 0.364 | Consistent |
| ApoB R3500Q | 27 | 27 / 0 / 0 | 0.000 | N/A‡ | 1.000 | Monomorphic |
| ApoE | 27 | 23 / 4 / 0 | 0.074 | 0.678 | 1.000 | Consistent |

*AA = homozygous wild-type; Aa = heterozygous; aa = homozygous variant. n = number of controls successfully genotyped for that locus. χ² = standard asymptotic chi-square goodness-of-fit test (df = 1). Exact p = exact test for HWE, the recommended method when expected genotype counts are small. Highlighted row indicates the locus with a statistically significant deviation from HWE under both methods. ‡ ApoB R3500Q was monomorphic among controls (all wild-type); the chi-square test is not computable when a genotype category is entirely absent, but the exact test remains valid and confirms consistency with HWE expectations*.

**Table S2**

| **Gene / Polymorphism** | **Genetic model** | **Raw p** | **Bonferroni p** | **BH-FDR q** | **Nominally significant (raw p<0.05)** |
| --- | --- | --- | --- | --- | --- |
| Factor V Leiden G1691A | GA vs GG (codominant) | 0.791 | 1.000 | 0.930 | No |
| Factor V Leiden G1691A | AA vs GG (codominant) | 0.020 | 0.640 | 0.160 | Yes |
| Factor V Leiden G1691A | Dominant | 0.089 | 1.000 | 0.310 | No |
| Factor V Leiden G1691A | Recessive | 0.017 | 0.544 | 0.160 | Yes |
| Prothrombin G20210A | GA vs GG (codominant) | 0.843 | 1.000 | 0.930 | No |
| Prothrombin G20210A | Dominant | 0.393 | 1.000 | 0.653 | No |
| MTHFR C677T | CT vs CC (codominant) | 0.218 | 1.000 | 0.537 | No |
| MTHFR C677T | TT vs CC (codominant) | 0.274 | 1.000 | 0.616 | No |
| MTHFR C677T | Dominant | 0.969 | 1.000 | 0.971 | No |
| MTHFR C677T | Recessive | 0.111 | 1.000 | 0.323 | No |
| MTHFR A1298C | het vs AA (codominant) | 0.929 | 1.000 | 0.971 | No |
| MTHFR A1298C | hom vs AA (codominant) | 0.097 | 1.000 | 0.310 | No |
| MTHFR A1298C | Dominant | 0.408 | 1.000 | 0.653 | No |
| MTHFR A1298C | Recessive | 0.067 | 1.000 | 0.306 | No |
| Factor V R2 (H1299R) | het vs WT (codominant) | 0.627 | 1.000 | 0.836 | No |
| Factor V R2 (H1299R) | Dominant | 0.716 | 1.000 | 0.916 | No |
| Factor XIII V34L | het vs WT (codominant) | 0.007 | 0.224 | 0.144 | Yes |
| Factor XIII V34L | Dominant | 0.009 | 0.288 | 0.144 | Yes |
| Beta-Fibrinogen -455G>A | het vs WT (codominant) | 0.327 | 1.000 | 0.616 | No |
| Beta-Fibrinogen -455G>A | Dominant | 0.489 | 1.000 | 0.745 | No |
| PAI-1 4G/5G | het vs WT (codominant) | 0.303 | 1.000 | 0.616 | No |
| PAI-1 4G/5G | hom vs WT (codominant) | 0.046 | 1.000 | 0.245 | Yes |
| PAI-1 4G/5G | Dominant | 0.142 | 1.000 | 0.379 | No |
| PAI-1 4G/5G | Recessive | 0.034 | 1.000 | 0.218 | Yes |
| GPIIIA HPA1 a/b | het vs WT (codominant) | 0.389 | 1.000 | 0.653 | No |
| GPIIIA HPA1 a/b | Dominant | 0.540 | 1.000 | 0.785 | No |
| ACE Insertion/Deletion | het vs WT (codominant) | 0.312 | 1.000 | 0.616 | No |
| ACE Insertion/Deletion | hom vs WT (codominant) | 0.971 | 1.000 | 0.971 | No |
| ACE Insertion/Deletion | Dominant | 0.089 | 1.000 | 0.310 | No |
| ACE Insertion/Deletion | Recessive | 0.586 | 1.000 | 0.815 | No |
| ApoE | het vs WT (codominant) | 0.841 | 1.000 | 0.930 | No |
| ApoE | Dominant | 0.841 | 1.000 | 0.930 | No |

*Raw p = unadjusted two-sided p-value. BH-FDR q = Benjamini-Hochberg false discovery rate–adjusted q-value.*
